# Serological evaluation of the effectiveness of reactive focal mass drug administration and reactive vector control to reduce malaria transmission in Zambezi Region, Namibia: Results from a secondary analysis of a cluster randomised trial

**DOI:** 10.1016/j.eclinm.2022.101272

**Published:** 2022-02-14

**Authors:** Lindsey Wu, Michelle S. Hsiang, Lisa M. Prach, Leah Schrubbe, Henry Ntuku, Mi-Suk Kang Dufour, Brooke Whittemore, Valerie Scott, Joy Yala, Kathryn W. Roberts, Catriona Patterson, Joseph Biggs, Tom Hall, Kevin K.A. Tetteh, Cara Smith Gueye, Bryan Greenhouse, Adam Bennett, Jennifer L. Smith, Stark Katokele, Petrina Uusiku, Davis Mumbengegwi, Roly Gosling, Chris Drakeley, Immo Kleinschmidt

**Affiliations:** a London School of Hygiene and Tropical Medicine, Faculty of Infectious Tropical Diseases, Department of Infection Biology, London, United Kingdom of Great Britain; b Department of Pediatrics, University of Texas Southwestern Medical Center, Dallas, TX, United States of America; c Malaria Elimination Initiative, Global Health Group, University of California San Francisco, San Francisco, CA, United States of America; d Department of Pediatrics, University of California San Francisco, San Francisco, CA, USA; e Division of Prevention Science, University of California San Francisco, San Francisco, CA, USA; f St. George's University of London, London, UK; g Division of Experimental Medicine, Department of Medicine, University of California San Francisco, San Francisco, CA, USA; h National Vector-Borne Diseases Control Programme, Namibia Ministry of Health and Social Services, Windhoek, Namibia; i Multidisciplinary Research Centre, University of Namibia, Windhoek, Namibia; j London School of Hygiene and Tropical Medicine, Faculty of Epidemiology and Population Health, Department of Infectious Disease Epidemiology, London, UK; k Research Council Collaborating Centre for Multi-Disciplinary Research on Malaria, School of Pathology, Wits Institute for Malaria Research, Faculty of Health Science, University of Witwatersrand, Johannesburg, South Africa; l Southern African Development Community Malaria Elimination Eight Secretariat, Windhoek, Namibia

**Keywords:** Malaria, Serology, Cluster randomised trials

## Abstract

**Background:**

Due to challenges in measuring changes in malaria at low transmission, serology is increasingly being used to complement clinical and parasitological surveillance. Longitudinal studies have shown that serological markers, such as Etramp5.Ag1, can reflect spatio-temporal differences in malaria transmission. However, these markers have yet to be used as endpoints in intervention trials.

**Methods:**

Based on data from a 2017 cluster randomised trial conducted in Zambezi Region, Namibia, evaluating the effectiveness of reactive focal mass drug administration (rfMDA) and reactive vector control (RAVC), this study conducted a secondary analysis comparing antibody responses between intervention arms as trial endpoints. Antibody responses were measured on a multiplex immunoassay, using a panel of eight serological markers of *Plasmodium falciparum* infection - Etramp5.Ag1, GEXP18, HSP40.Ag1, Rh2.2030, EBA175, *Pf*MSP1_19_, *Pf*AMA1, and *Pf*GLURP.R2.

**Findings:**

Reductions in sero-prevalence to antigens Etramp.Ag1, *Pf*MSP1_19_, Rh2.2030, and *Pf*AMA1 were observed in study arms combining rfMDA and RAVC, but only effects for Etramp5.Ag1 were statistically significant. Etramp5.Ag1 sero-prevalence was significantly lower in all intervention arms. Compared to the reference arms, adjusted prevalence ratio (aPR) for Etramp5.Ag1 was 0.78 (95%CI 0.65 – 0.91, *p* = 0.0007) in the rfMDA arms and 0.79 (95%CI 0.67 – 0.92, *p* = 0.001) in the RAVC arms. For the combined rfMDA plus RAVC intervention, aPR was 0.59 (95%CI 0.46 – 0.76, *p* < 0.0001). Significant reductions were also observed based on continuous antibody responses. Sero-prevalence as an endpoint was found to achieve higher study power (99.9% power to detect a 50% reduction in prevalence) compared to quantitative polymerase chain reaction (qPCR) prevalence (72.9% power to detect a 50% reduction in prevalence).

**Interpretation:**

While the observed relative reduction in qPCR prevalence in the study was greater than serology, the use of serological endpoints to evaluate trial outcomes measured effect size with improved precision and study power. Serology has clear application in cluster randomised trials, particularly in settings where measuring clinical incidence or infection is less reliable due to seasonal fluctuations, limitations in health care seeking, or incomplete testing and reporting.

**Funding:**

This study was supported by Novartis Foundation (A122666), the Bill & Melinda Gates Foundation (OPP1160129), and the Horchow Family Fund (5,300,375,400).

**Research in Context**

**Evidence before this study**

Numerous serological studies across sub-Saharan Africa have found that malaria-specific antibody responses are highly correlated with malaria transmission. Serology is increasingly being used to complement traditional malaria surveillance data in settings where clinical or parasitological measures may be less reliable due to fluctuations in parasite densities, limitations in health care seeking, or incomplete testing and reporting. Newly identified serological markers associated with recent malaria exposure hold promise as measures of malaria incidence. In previous longitudinal cohort studies in The Gambia, Etramp5.Ag1 has been shown to be a discriminatory serological marker capable of detecting spatio-temporal differences in malaria transmission. However, these markers have never been formally used as endpoints in a malaria cluster randomised trial. On 26 July 2021, based on a PubMed search for original articles with no restrictions on language or time period, using the search terms “Plasmodium falciparum OR malaria” AND “serology” AND “cluster randomized trial”, no studies were found that assess antibody responses as an endpoint in a cluster randomised trial evaluating interventions to reduce malaria transmission.

**Added value of this study**

This study is the first application of serological endpoints in a malaria cluster randomised trial. Using a multiplexed immunoassay, a panel of sero-incidence markers of recent malaria exposure were used to evaluate the effectiveness of reactive focal mass drug administration (rfMDA) and reactive focal vector control (RAVC) compared to reactive case detection (standard of care) to reduce malaria transmission. Cluster-level antibody responses were significantly lower in all intervention arms compared to control, and effect sizes were measured with greater study power than other trial endpoints such as quantitative polymerase chain reaction (qPCR) parasite prevalence.

**Implications of all the available evidence**

The findings from this study, together with ongoing innovations in assay design and multi-disease platforms, illustrate the potential application of serological markers as endpoints in cluster randomised trials. The use of serological endpoints can help achieve trial efficiencies, such as reduced sample size, particularly in low transmission settings or multi-intervention trials where measuring differences between study arms may be challenging with clinical or parasitological endpoints alone.

Alt-text: Unlabelled box

## Introduction

In elimination settings, cluster randomised trials measuring changes in malaria transmission face a number of challenges, particularly when estimating clinical incidence and parasite prevalence as endpoints. At low transmission, differences between study arms can be subtle, while between-cluster variation within study arms can be large.^1^ Passive surveillance based on routine health systems often do not capture asymptomatic individuals or may under-estimate clinical incidence in areas where care-seeking is low.^2^, ^3^, ^4^, ^5^ While active surveillance measuring parasite prevalence can improve the detection of infections in the wider community, fluctuations in parasite density throughout a season can result in a sizeable number of infections becoming undetectable at any given time.^6^^,^^7^ For these reasons, measuring clinical incidence and parasite prevalence as trial endpoints can be imprecise, making it difficult to design studies with adequate sample sizes or study power.^8^

Serological methods are increasingly being used alongside clinical and parasitological metrics for surveillance. Studies in moderate to low transmission regions across sub-Saharan Africa, including Tanzania,^9^ Equatorial Guinea,^10^ South Africa^11^ and The Gambia,^12^ have found that malaria-specific antibody responses are highly correlated with parasitological endpoints. Serological assays can be a particularly useful tool in settings where parasite densities commonly fall below the detection limit of other diagnostics such as microscopy or RDTs.^13^, ^14^, ^15^ In some areas, serology has also been used to confirm interruption of malaria transmission.^16^^,^^17^

Namibia is one of a number of southern African countries targeting malaria elimination.^18^^,^^19^ Recently, however, areas of northern Namibia have experienced periodic spikes in incidence,^20^ creating challenges for elimination efforts. In 2017, a cluster randomised control trial was conducted in Zambezi Region, Namibia, evaluating reactive focal mass drug administration (rfMDA) and reactive vector control (RAVC) as new approaches to reduce malaria transmission.^21^ The study presented here is an extended trial analysis assessing cluster-level antibody responses to a panel of serological markers previously shown to be associated with parasitological measures of malaria.^12^^,^^22^

Most serological studies for malaria have monitored historical trends based on long-lived antibody responses rather than with sero-incidence markers associated with short-lived antibody responses. Sero-incidence markers aim to measure recent exposure or detect rapid changes in transmission (e.g., over periods of 1 to 5 years). Recent studies analysing cohorts in Uganda and Mali have identified several serological markers that are predictive of clinical malaria in the previous year.^23^ Etramp5.Ag1 in particular has been shown to be a discriminatory sero-incidence measure between geographical regions and transmission seasons in The Gambia.^12^^,^^22^ However, serological markers of malaria exposure have rarely been used as outcome measures for cluster randomised trials. Etramp5.Ag1 belongs to the early transcribed membrane protein family. These proteins have been shown to localise in the parasitophorous vacuole membrane where the parasite resides while inside the erythrocyte and hepatocyte, and may play a role in mediating *Plasmodium*-host cell interaction.^24^

Using a multiplexed bead-based assay and samples from the endline cross-sectional survey of the rfMDA/RAVC trial, this study sought to examine whether leading serological candidates for measuring recent malaria infection can provide secondary evidence of intervention effects in a cluster randomised trial. Serology was compared against primary trial endpoints malaria case incidence and parasite infection prevalence. This study aims to (1) assess whether reduced antibody responses for the candidate markers evaluated would be observed in intervention arms due to relative reductions in malaria transmission, and (2) estimate a number of trial design parameters based on serology as a trial endpoint, including inter-cluster coefficient of variation and trial sample sizes, to assess whether improved study power and trial efficiencies can be achieved.

## Methods

***Data and study design.*** The study was an analysis of data from an open label cluster randomised controlled trial with a 2 × 2 factorial study design (Supplementary Figure S1) with four study arms receiving the following interventions:

1. **RACD (standard of care control arm):** rapid diagnostic testing and treatment of positives with artemether-lumefantrine (AL) and single dose primaquine of individuals residing within a 500 m radius of a recent passively detected index case
2. **rfMDA:** presumptive treatment with artemether-lumefantrine (AL) of individuals residing within a 500 m radius of a recent passively detected index case
3. **RAVC and RACD combined:** indoor residual spraying (IRS) using pirimiphos-methyl, administered to households of individuals residing within a 500 m radius of a recent passively detected index case, plus standard of care RACD as described above
4. **rfMDA and RAVC combined:** indoor residual spraying (IRS) using pirimiphos-methyl, administered to households of individuals residing within a 500 m radius of a recent passively detected index case, plus rfMDA as described above

All clusters received routine annual IRS before the start of the malaria season using dichloro-diphenyl-trichloroethane (DDT) conducted as part of standard malaria control activities by the Namibian Ministry of Health and Social Services (MoHSS).

The trial was conducted in Zambezi Region, Namibia, from January to December 2017, within the catchment areas for 11 health facilities. 56 enumeration areas (EAs) in the study area that met the inclusion criteria were selected and randomly allocated to one of four arms using restricted randomisation. Restriction criteria included mean annual incidence in 2013 and 2014, population size, population density, and mean distance from the household to a health-care facility. The primary outcome of the main study was cumulative incidence of passively detected malaria. Secondary outcomes included infection prevalence, intervention coverage, refusal rates, adverse events, and adherence to drug regimen. Details of the study are reported on ClinicalTrials.gov: NCT02610400^25^ and described in Medzihradsky et al., 2018.^26^ The clinical and parasitological results of the trial are reported in Hsiang et al.^21^

An endline cross-sectional survey was conducted as part of the trial at the end of the malaria season from May to August 2017 to measure infection prevalence by qPCR and sero-prevalence. Out of a total of 1333 index cases reported during the trial, only 8 cases were reported from September to December 2017 after the endline survey was conducted. Within each of the 56 clusters, 25 households were randomly sampled for inclusion in the cross-sectional survey. The survey was designed to have 80% power to detect a minimum decrease in sero-prevalence of 5.3% for rfMDA vs. RACD and RAVC vs non-RAVC, assuming 10% sero-prevalence in RACD arms (sample size of 6300 sampled and 5040 enroled, with 2520 in the two rfMDA arms, 2520 in the two RACD arms, and 2520 in the two RAVC arms and 2520 in the non-RAVC arms).^26^ All residents older than six months who slept in the household at least three nights per week in the previous four weeks were eligible for inclusion in the cross-sectional survey. For consenting individuals, blood samples were collected by finger prick on dry blood spot (DBS) filter paper (Whatman 3 Corporation, Florham Park, NJ, USA) and 250 μl of whole blood in BD Microtainer® tubes with EDTA additive (Becton, Dickinson and Corporation, Franklin Lakes, NJ, USA) for molecular and serological analysis. Individuals, both symptomatic and asymptomatic, with positive RDT results were treated with AL and single dose primaquine according to national guidelines.^27^ To avoid the introduction of bias due to timing of sample collection, all study arms were sampled in parallel during the three-month cross-sectional survey. The rate of enrolment was monitored and standardised between study arms to prevent imbalances in sampling. Based on a mixed-methods study to assess acceptability of the trial interventions through key informant interviews, most non-participants indicated that they were unavailable during the intervention as the reason for not participating. However, some expressed a low malaria-risk perception and some community members indicated that refusal was due to disagreement with presumptive treatment.^28^

This study adheres to CONSORT guidelines for cluster randomised trials and STROBE guidelines for cross-sectional surveys.

***Ethical approval and consent.*** The trial received ethical approval from the Namibia MoHSS (17/3/3), and the Institutional Review Boards of the University of Namibia (MRC/259/2017), University of California San Francisco (15–17,422) and London School of Hygiene & Tropical Medicine (10,411). Written informed consent was obtained from individual participants for rfMDA or RACD, and from heads of households (≥18 years of age) for RAVC. A parent or guardian was required to provide written informed consent for children younger than 18 years receiving rfMDA or RACD, and written consent for receiving these interventions was also obtained from children aged 12–17 years.

### Laboratory procedures

Human plasma from whole blood samples were prepared and tested on the Luminex assay platform using procedures described by Wu et al.^29^ Plasma samples from study participants were prepared from 250 μl of whole blood collected in BD Microtainer tubes with EDTA additive. Two sets of positive controls were used based on pooled sera from 100 hyper-immune Tanzanian individuals and a WHO malaria reference lyophilised serum reagent (NIBSC 10–198).^30^ Plasma samples from European malaria-naïve adults were used as negative controls. Two wells on each plate containing only antigen-coupled beads and sample buffer were included to measure background signal.

***Antigen selection and design.*** A subset of eight antigens (Etramp5.Ag1, GEXP18, HSP40.Ag1, Rh2.2030, EBA175, *Pf*MSP1_19_, *Pf*AMA1, *Pf*GLURP.R2) were selected from an initial screen of 856 candidates on an in vitro transcription and translation (IVTT) protein microarray based on their correlation with clinical and parasitological endpoints in previous studies.^12^^,^^22^ Antigens were expressed in *Escherichia coli* (*E.coli*) as glutathione S-transferase (GST)-tagged fusion proteins, except for *Pf*AMA1 expressed in Pichia pastoris as a histidine-tagged protein. Non-malaria reactivity against GST-tagged fusion proteins were assessed using IgG responses to GST-coupled beads, and samples with greater than 1000 median fluorescence intensity (MFI) were excluded from analyses due to high non-specific IgG response. A full description of laboratory methods, including antigen constructs, expression platform, coupling conditions and data standardisation are detailed further in Wu et al^29^ and summarised in Supplementary Table S1. Antigens were classified as long- or short-term markers of malaria infection based on their association with cumulative malaria exposure (*Pf*MSP1_19_, *Pf*AMA1, *Pf*GLURP.R2)^9^^,^^31^^,^^32^ or recent malaria exposure (Etramp5.Ag1, GEXP18, HSP40.Ag1, Rh2.2030, EBA175).^23^^,^^33^

### Statistical analyses

Serological responses were used as endpoints to assess (Supplementary Figure S1):

1. rfMDA vs. RACD (with or without RAVC)
2. RAVC vs No RAVC (with either RACD or rfMDA)
3. rfMDA plus RAVC vs. RACD only

In line with the primary trial analysis, reported by Hsiang et al.,^21^ a modified intention-to-treat analysis was conducted, which adjusted at the EA-level for baseline incidence in 2016, intervention response time, proximity to a Namibia MoHSS co-intervention (within 500 m of a village receiving concomitant MoHSS active case detection or IRS), index case coverage (the proportion of eligible index cases covered by an intervention), and the household coverage of the target population (the proportion of eligible individuals or households within an intervention event area triggered by an index case that actually received the intervention).

Serological responses were measured as median fluorescence intensity (MFI) values, and standardised to account for between plate variation using a loess normalisation method described in Wu et al.^34^ Sero-positivity values for all samples were assigned according to MFI thresholds defined by the mean and three standard deviations of a pool of 71 malaria naïve blood donors used as negative controls.^12^^,^^29^ For antigens associated with cumulative exposure (*Pf*MSP1_19_, *Pf*AMA1, and *Pf*GLURP.R2) ,^9^^,^^31^^,^^32^ individuals previously exposed but not infected for 5 or more years may still have higher average antibody responses^35^^,^^36^ than malaria-naïve donors. For these markers, sero-positivity MFI thresholds were defined as the mean and two standard deviations of the lower component of a Gaussian mixture model fit to antibody responses of the endemic population. Serological responses were then assessed to compare the intervention study arms.

***Sero-prevalence.*** Sero-prevalence of population antibody responses to each antigen was estimated using generalised linear models (GLM) with log link, binomial family, generalised estimating equations (GEE) allowing for within cluster correlation and assessed according to the three interventions described above. Results are presented as unadjusted mean sero-prevalence by intervention and study arm and as unadjusted and adjusted sero-prevalence ratios, where the denominator is mean sero-prevalence of clusters in the reference study arm (RACD only). Additionally, the combined sero-prevalence to any short-term marker (i.e. sero-positivity to at least one of Etramp5.Ag1, GEXP18 or HSP40.Ag1) as an overall measure of sero-incidence was also estimated to assess whether sero-positivity to multiple markers would result in an enhanced comparison between interventions. To assess whether timing of sample collection impacted results, visit day was included as a covariate in initial logistic regression analysis. No significant effect was observed for any antigen, so day of sample collection was excluded as a variable from final analysis.

***Antibody acquisition.*** Dichotomisation of data into positive and negative categories, the basis for estimating sero-prevalence, can lead to some loss of sensitivity in detecting changes in malaria transmission.^10^ Therefore, changes in the magnitude of antibody responses were also assessed using antibody acquisition models, which estimates the geometric mean MFI by age^22^ for each antigen and cluster. Using this model fit, the total area under the antibody acquisition curve, referred to in this analysis as the AUC value, represents the cumulative antibody response across all ages. AUC values are estimated using an antibody acquisition model fit extrapolated to a standardised age range of 1–90 years to account for between-cluster variation in age range. The effect of the intervention on mean log AUC across clusters was assessed linear regression, inverse-weighted by the 95% CI of the cluster AUC values, and results are presented as unadjusted and adjusted AUC ratios, where the denominator is mean AUC value of clusters in the reference study arm.

For both sero-prevalence and antibody acquisition, regression analysis tested the effect of each intervention independently as well as with an interaction between rfMDA and RAVC, allowing assessment of the effect of each intervention singly (rfMDA or RAVC) or in combination (rfMDA plus RAVC). This allows interpretation of whether the combination of rfMDA and RAVC interventions were simply additive, synergistic, or antagonistic (i.e., whether targeting both the human and mosquito reservoirs of infection has a biological advantage in reducing malaria).

***Between cluster coefficient of variation and sample size calculations.*** To assess how trial sample sizes might be affected by using serological outcome measures for the design of a study, we estimated the between cluster coefficient of variation, k,^37^ for sero-prevalence to Etramp5.Ag1 (the best performing individual marker) to compare these to corresponding values of k for qPCR. Analysis was limited to RACD only arms as a proxy for baseline antibody patterns. The required number of clusters per arm, c,^37^ was calculated to demonstrate a 50% or greater reduction in sero-prevalence in clusters receiving either RAVC or rfMDA alone and a 75% reduction in clusters receiving the combined intervention (rfMDA plus RAVC), compared to RACD only as the control. These percentages were the expected reductions in clinical incidence upon which the main study was designed. Sample size (number of clusters per study arm) was estimated for a range of cluster sizes, assuming a desired study power of 80% and a 5% two-sided significance level. As a comparator, the coefficient of variation and minimum sample sizes were also calculated using qPCR prevalence as the endpoint.

***Role of funding sources.*** The funders had no role in study design, data collection and analysis, decision to publish, or preparation of the manuscript.

## Results

***Study participant characteristics and intervention implementation and coverage.*** A total of 4361 individuals were enroled in the end-line cross-sectional survey, of which 4164 samples were available for serological processing. After excluding individuals with high responses to GST (an expression tag present on most protein constructs) to avoid the influence of non-malaria specific antibody response, a total of 3657 samples were available for final analysis. PCR prevalence was not significantly different between individuals included for serological analysis (3.05% 95%CI 2.48 – 3.62) compared to individuals excluded from serological analysis (3.97% 95%CI 2.66 – 5.28). Age and gender distribution were balanced across study arms, with the proportion of individuals aged 15 years and older ranging from 52.9% (396 out of 749 in rfMDA plus RAVC arm) to 55.5% (508 out of 915 in the rfMDA only arm) and proportion of females ranging from 53.4% (400 out of 749 in the rfMDA plus RAVC arm) to 56.1% (555 out of 990 in the RACD only arm) (Supplementary Table S2). Participants that reported sleeping outdoors in the previous two weeks ranged from 5.3% (52 out of 990 in the RACD only arm) to 7.0% (64 out of 915 in the rfMDA only arm) and participants that reported sleeping under a bed net the previous night ranged from 18.4% (168 out of 915 in the rfMDA only arm) to 26.5% (198 out of 749 in the rfMDA plus RAVC arm) (Supplementary Table S2). Intervention coverage by index case and target population and proximity to MoHSS co-intervention were reported previously.^21^ Briefly, mean target population or household intervention coverage ranged from 86.4% to 93.3% , and between 43.4% and 61.8% of households (by study arm) were within 500 m of a village that received concomitant additional interventions carried out by the Namibia MoHSS.

**Figure 1:**
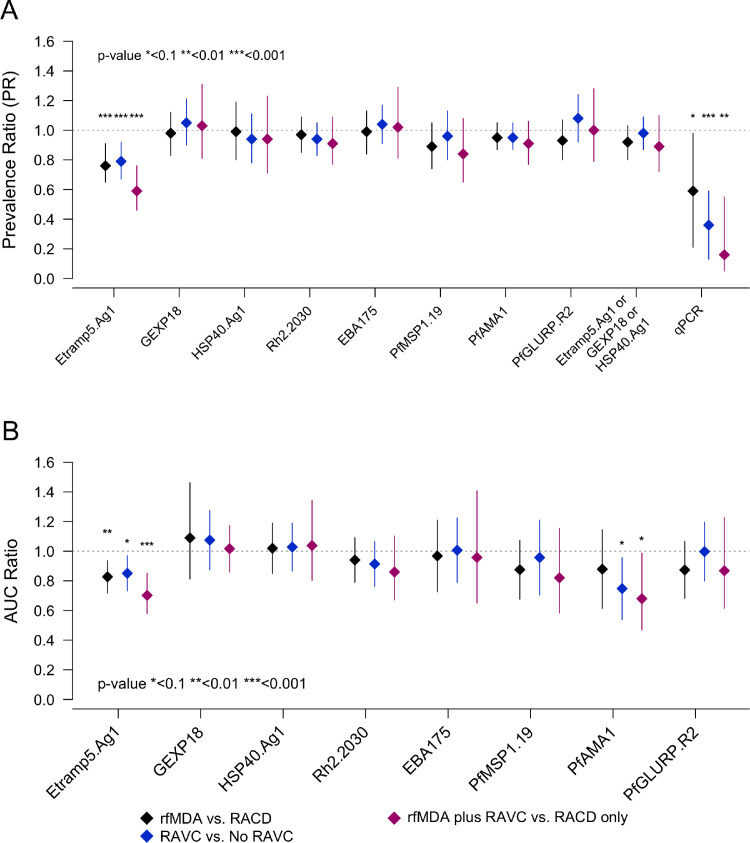
Sero-prevalence ratio, qPCR prevalence ratio, and AUC ratio by antigen and intervention. (A) Adjusted prevalence ratios are shown for rfMDA vs RACD (black), RAVC vs. No RAVC (blue) and rfMDA plus RAVC vs. RACD only (magenta). (B) Adjusted ratio of log AUC values are shown for rfMDA vs RACD (black), RAVC vs. No RAVC (blue) and rfMDA plus RAVC vs. RACD only (magenta). All values are adjusted for EA incidence in 2016, proportion of EA index cases covered, proportion of target population covered, median time to intervention, and distance from villages receiving an MoHSS intervention.

***Sero-prevalence by study arm.*** While lower sero-prevalence was observed in the rfMDA and RAVC arms for a number of antigens, these differences were consistently significant for Etramp5.Ag1, which exhibited the most pronounced differences between study arms. Sero-prevalence to Etramp5.Ag1 was significantly lower in all intervention arms compared to the reference arms (Figure 1A, Table 1, Supplementary Table S4). In the rfMDA arms (28 clusters), unadjusted mean Etramp5.Ag1 sero-prevalence was 21.7% (95% CI 18.7 – 24.7) compared to 27.2% (95%CI 24.3 – 30.1) in the RACD reference arms (27 clusters), adjusted prevalence ratio (aPR) 0.78 (95%CI 0.65 – 0.91, *p* < 0.001). Mean Etramp5.Ag1 sero-prevalence for RAVC arms (28 clusters) was 22.0% (95%CI 19.1 – 25.0) vs 26.8% (95%CI 23.9 – 29.8) in the non-RAVC arms (27 clusters), aPR 0.79 (95%CI 0.67 – 0.92, *p* = 0.001). The largest effect was observed in the combined rfMDA plus RAVC arms (14 clusters), where mean Etramp5.Ag1 sero-prevalence was 17.8% (95%CI 13.4 – 22.3) compared to 28.1% (95%CI 23.7 – 32.6) in the RACD only arms (13 clusters), aPR 0.59 (95%CI 0.46 – 0.76, *p* < 0.001). There was no strong statistical evidence that the rfMDA and RAVC interventions acted synergistically to reduce sero-prevalence (interaction coefficient 0.75 95%I 0.56 – 1.02, *p* = 0.067). Population antibody responses varied by antigen, with sero-prevalence in the RACD only arm ranging from 22.9% (95%CI 19.0 – 26.7) for HSP40.Ag1 and 28.1% (95%CI 23.7 – 32.6) for Etramp5.Ag1 to 48.3% (95%CI 44.2 – 52.5) for *Pf*AMA1 (Supplementary Figure S2, Table S3).

**Table 1:** Etramp5.Ag1 sero-prevalence ratio by intervention. Mean sero-prevalence and sero-prevalence ratios are estimated using generalised linear models by intervention (with log link, binomial family, and GEE with clustering at EA-level). Prevalence ratios are adjusted for EA incidence in 2016, proportion of EA index cases covered, proportion of target population covered, median time to intervention, and distance from villages receiving an MoHSS intervention. Unadjusted model includes an interaction coefficient of 0.79 (95% CI 0.55 – 1.16, *p* = 0.23) and adjusted model includes an interaction coefficient of 0.75 (95% CI 0.56 – 1.02), *p* = 0.067.

			Unadjusted			Adjusted	
	Number of individuals	Number of clusters	Mean prevalence (95% CI)	Prevalence ratio (95% CI)	p-value	Prevalence ratio (95% CI)	p-value
Human reservoir							
RACD (reference)*	1993	27	0.27 (0.24 – 0.30)	1.00	–	1.00	–
rfMDA*	1664	28	0.22 (0.19 – 0.25)	0.78 (0.64 – 0.93)	0.0038	0.78 (0.65 – 0.91)	0.0007
Mosquito reservoir							
No RAVC (reference)†	1905	27	0.27 (0.24 – 0.30)	1.00	–	1.00	**–**
RAVC†	1752	28	0.22 (0.19 – 0.25)	0.85 (0.70 – 1.00)	0.057	0.79 (0.67 – 0.92)	0.001
Human and mosquito reservoir							
RACD only (reference)	990	13	0.28 (0.24 – 0.33)	1.00	–	1.00	**–**
rfMDA plus RAVC	749	14	0.18 (0.13 – 0.22)	0.65 (0.49 – 0.87)	0.0034	0.59 (0.46 – 0.76)	<0.0001

⁎ With or without RAVC.

† With either RACD or rfMDA.

While the relative reduction in qPCR prevalence was greater, trends in effect size between study arms measured with qPCR in the main study were similar to serology, as shown in Figure 1A, where the qPCR aPR was 0.59 (95%CI 0.21 – 0.98, *p* = 0.039) for rfMDA (28 clusters) versus RACD (27 clusters), 0.36 (95%CI 0.13 – 0.59, *p*<0.0001) for RAVC (28 clusters) versus non-RAVC (27 clusters), and 0.16 (95%CI 0.05 – 0.55, *p* = 0.004) for rfMDA plus RAVC (14 clusters) versus RACD only (13 clusters) (Supplementary Table S5). Sero-prevalence to a combination of sero-incidence markers (Etramp5.Ag1, GEXP18, and HSP40.Ag1) was also assessed, but no significant differences between study arms were observed. The distribution of cluster-level sero-prevalence by study arm is shown in Supplementary Figure S3.

***Antibody acquisition by study arm.*** Analysis of cluster-level Etramp5.Ag1 AUC values based on continuous antibody response confirmed similar effects between study arms (Figure 1B, Table 2, Supplementary Figure S4, Supplementary Table S6). The adjusted AUC ratio of rfMDA (28 clusters) was 0.83 (95%CI 0.72 – 0.94, *p* = 0.0019) relative to RACD (27 clusters), and for RAVC (28 clusters), the AUC ratio was 0.85 (95%CI 0.73 – 0.97, *p* = 0.014) relative to non-RAVC (27 clusters). The AUC ratio for rfMDA plus RAVC combined (14 clusters) was 0.70 (95% CI 0.58 - 0.85, *p* = 0.00032) relative to RACD only (13 clusters). Differences in AUC values between study arms were also observed for several other antigens (Figure 1B) but may be due to multiple testing across antigens as this was not observed for all interventions and most results were not statistically significant. The distribution of cluster-level AUC values is shown by study arm in Supplementary Figure S5 and the antibody acquisition fit individually for each EA is shown in Supplementary Figure S6.

**Table 2:** Etramp5.Ag1 Area under the antibody acquisition curve (AUC) by intervention. Reference arms are clusters in the RACD, non-RAVC, or RACD only arms. Ratio of log AUC values in the intervention vs reference arms are estimated using generalised linear models (log link, gaussian family, and GEE for clustering at the EA-level) and adjusted for EA incidence in 2016, proportion of EA index cases covered, proportion of target population covered, median time to intervention, and distance from villages receiving an MoHSS intervention. Unadjusted model includes an interaction coefficient of 0.85 (95% CI 0.65 – 1.13, *p* = 0.27) and adjusted model includes an interaction coefficient of 0.83 (95% CI 0.64 – 1.07), *p* = 0.15.

		Unadjusted			Adjusted	
	Number of clusters	Mean AUC value (95%CrI)	AUC ratio (95%CrI)	p-value	AUC ratio (95%CrI)	p-value
Human reservoir						
RACD (reference)*	27	34,595 (31,496 – 37,693)	1.00	–	1.00	–
rfMDA*	28	28,337 (25,429 – 31,244)	0.82 (0.71 – 0.93)	0.0015	0.83 (0.72 – 0.94)	0.0019
Mosquito reservoir						
No RAVC (reference)†	27	33,211 (30,074 – 36,348)	1.00	–	1.00	**–**
RAVC†	28	29,382 (26,516 – 32,248)	0.88 (0.76 – 1.00)	0.060	0.85 (0.73 – 0.97)	0.014
Human and mosquito reservoir						
RACD only (reference)	13	35,449 (31,307 – 40,138)	1.00	–	1.00	**–**
rfMDA plus RAVC	14	25,553 (21,060 – 31,005)	0.72 (0.59 – 0.87)	0.0009	0.70 (0.58 – 0.85)	0.00032

⁎ With or without RAVC.

† With either RACD or rfMDA.

**Figure 2:**
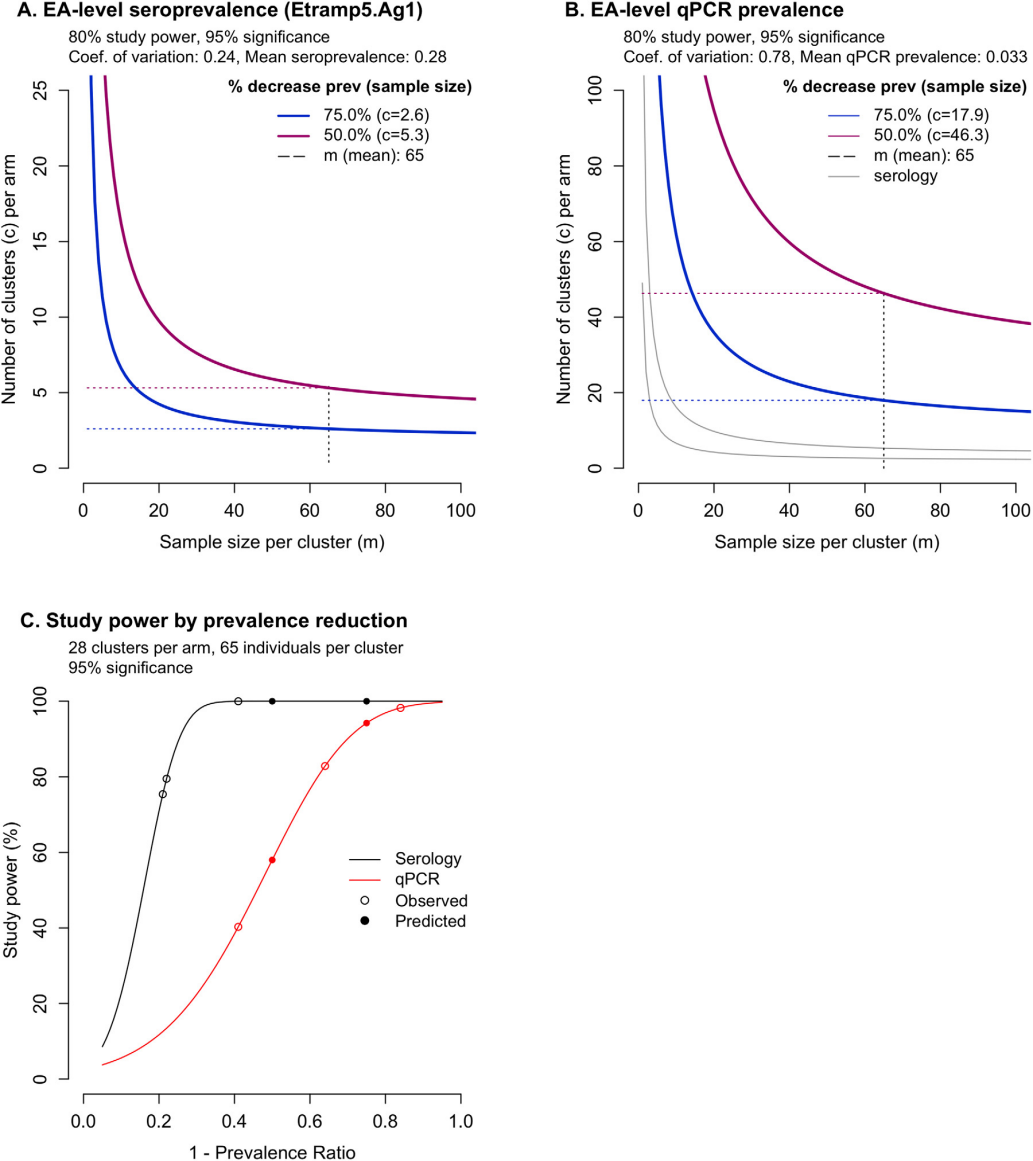
Coefficient of variation, k, and number of clusters per arm, c, using serology compared to qPCR as a trial endpoint. Number of clusters per arm is estimated for serology (A) and qPCR (B) based on predicted decrease in prevalence of 75% in clusters receiving the combined intervention rfMDA plus RAVC arm (blue) and 50% in clusters receiving either rfMDA or RAVC alone (magenta). Mean cluster sample size, m (mean), is indicated by the dotted vertical black line, and the associated number of clusters required indicated by the horizontal dotted lines. Change in study power by relative reduction in prevalence (C) is shown for serology (black) and qPCR (red), with study power for predicted and observed relative reduction in prevalence indicated by filled and empty circles.

***Serology-based sample size and study power estimation***. For a range of cluster sizes, the number of clusters per arm was estimated to detect an effect size of either a 75% or 50% reduction in sero-prevalence with 80% study power and a 5% significance level (alpha = 5%), assuming a baseline sero-prevalence of 0.28 (mean sero-prevalence RACD only arm, Table 1). These percentages were used because the main study was powered to detect an expected 50% reduction in clinical incidence in clusters receiving either the rfMDA or RAVC alone and a 75% reduction in clusters receiving the combined intervention (rfMDA plus RAVC). The estimated sero-prevalence coefficient of variation between clusters, k, in RACD only clusters was 0.24, compared to a qPCR prevalence coefficient of variation of 0.78 (Figure 2A and 2B). Assuming an average cluster sample of 65 individuals (based on the mean number of respondents per cluster in the cross-sectional survey), the minimum sample size required to observe a 75% reduction in sero-prevalence (between rfMDA plus RAVC vs RACD only) was 2.6 clusters per arm, while 5.3 clusters per arm would be required to observe a 50% reduction in sero-prevalence (between either rfMDA vs RACD or RAVC vs. no RAVC).

Alternatively, for the trial as designed with 28 clusters per arm to compare the two main effects (rfMDA vs RACD, and RAVC vs no RAVC), and 14 clusters per arm to compare the combination rfMDA plus RAVC vs RACD only, there would have been >99.9% power to detect reductions in seroprevalence of 50% and 75% respectively for each of the comparisons (i.e., virtually no chance of a type 2 error) (Figure 2C).

By contrast, assuming a baseline qPCR prevalence of 0.033 (mean prevalence in RACD only clusters), a minimum of 17.9 and 46.3 clusters per arm would be required to detect a decrease of 75% and 50% qPCR prevalence, respectively. Based on modelled relationship between log odds of sero-positivity and log odds of qPCR positivity (Supplementary Materials equation 1), a qPCR prevalence of 0.033 translates to Etramp5.Ag1 sero-prevalence of 0.25 (95% CrI 0.23 – 0.28), aligned with the observed baseline sero-prevalence in the RACD only arms noted above. As noted above, the effect sizes observed for qPCR were greater than for serology (84% vs 41% relative reduction in prevalence for the combined rfMDA plus RAVC arms), and this should be considered when comparing the expected study power of these metrics for different settings or studies.

**Figure 3:**
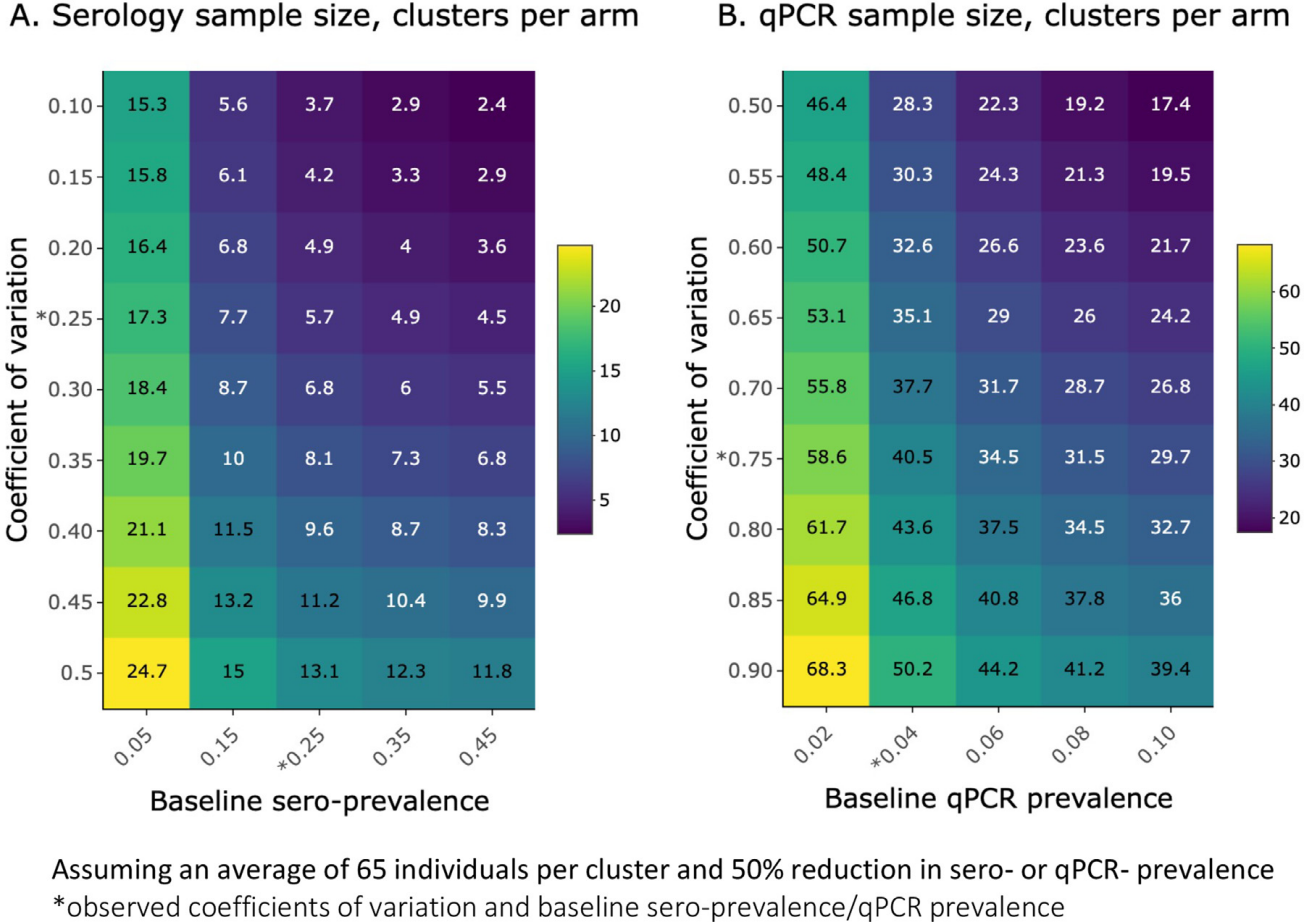
Number of clusters per arm, c, for a range of baseline prevalence and coefficient of variation values. Heatmaps show the number of clusters per arm required for a range of coefficient of variation values and sero-prevalence (A) or qPCR prevalence (B), assuming an average of 65 individuals per cluster and 50% reduction in sero- or qPCR- prevalence. Observed coefficients of variation and baseline sero- and qPCR-prevalence are indicated by asterisks.

Sensitivity analysis of the effect of baseline prevalence and coefficient of variation on required sample size was explored for both serology and qPCR endpoints (Figure 3A and 3B). For serology, based on baseline sero-prevalence ranging from 0.05 to 0.45 and coefficient of variation values between 0.1 to 0.5, the estimated sample size was between 2.4 and 24.7 clusters per arm. For qPCR, based on baseline qPCR prevalence from 0.02 to 0.10 and coefficient of variation values between 0.5 to 0.9, estimated sample sizes ranged from 17.4 to 68.3 clusters per arm. When using 28 clusters per study arm, while there is 94% study power to detect a 75% reduction in qPCR prevalence, this study power drops to 58% when trying to detect a 50% reduction (Figure 3C).

## Discussion

This study sought to assess the application of serological markers for malaria exposure in the context of a cluster randomised trial, which was originally analysed using clinical incidence and qPCR prevalence as outcomes. Previous analysis showed that the introduction of rfMDA and RAVC, independently and in combination, had a significant effect on malaria clinical incidence and qPCR prevalence, after adjusting for factors that could not be balanced between study arms.^21^ This analysis was replicated and resulted in similar observations when assessing the efficacy of the interventions using the serological marker Etramp5.Ag1; rfMDA and RAVC were shown to be associated with significantly reduced antibody responses as independent and combined interventions. The intervention effects on antibody responses were detected using both binary and continuous antibody measurements.

The use of Etramp5.Ag1 as a sero-incidence marker to evaluate trial outcomes was found to be comparable to the use of qPCR parasite prevalence, with the added benefit of increased precision in the measure of effect size (a standard error for sero-prevalence ratio of only 0.06 compared to a standard error of 0.19 for qPCR prevalence ratio). This is likely due to smaller seasonal fluctuations in population antibody responses compared to variations in parasitaemia that can affect detection by molecular assays. In previous studies in The Gambia, Etramp5.Ag1 antibody responses was found to persist for several months following the transmission season, before waning prior to transmission season.^22^ This suggests that serology is a more temporally stable measurement of malaria exposure throughout the transmission season but is still able to detect short-term changes occurring over the course of less than a year. This improved measurement consistency is reflected in several ways: higher overall levels of sero-prevalence, marked differences between study arms, combined with reduced between-cluster coefficient of variation for sero-prevalence (which was two-thirds lower than the coefficient of variation for qPCR prevalence). These can translate to significantly improved study power to detect fine-scale changes in transmission with smaller sample sizes and higher precision in effect estimates. In multi-intervention studies, smaller effects are likely envisaged between study arms. For example, a number of trials are currently comparing the use of MDA with or without ivermectin,^38^ as well as the use of the RTS,S malaria vaccine combined with seasonal malaria chemoprevention (SMC).^39^ These studies will likely be evaluated in the context of standard malaria control interventions already in use and detecting subtle differences may be challenging.

Serological endpoints may be particularly relevant in the design of cluster randomised trials in low transmission settings with limitations in study power.^8^ In these areas, rates of clinical incidence or infection prevalence may be very low or close to zero and would require prohibitively large sample sizes to detect intervention effects. This is highlighted in this study in Namibia, where all clusters measuring a qPCR prevalence or clinical incidence of zero still had detectable sero-positive individuals (Supplementary Figure S7). Serology may also serve as a useful secondary endpoint to other measures of infection such as PCR. As with all surveillance diagnostics, imperfect sensitivity and specificity will impact the interpretation of prevalence estimates.^40^ Due to the persistence of antibodies after parasite clearance, serological measures may have a reduced specificity for detecting current infection, but conversely have an increased sensitivity for detecting cumulative infections over a period of time relative to more static measures such as PCR. Serology may be particularly useful for detecting recent history of malaria exposure over months or years that could only be detected with PCR prevalence using multiple cross-sectional surveys.

Ongoing research to develop new serological markers into point-of-care devices may enable a new method for rapid field-based malaria surveillance.^41^ This is increasingly important in settings such as Namibia to monitor for potential outbreaks and to prevent reintroduction of malaria infection following elimination. For Namibia, serological surveillance could be beneficial given the fluctuations in malaria incidence in recent years. Due to frequent population movement from neighbouring countries, especially along the Angolan and Zambian borders, low to moderate transmission has been found to persist and receptivity remains high.^42^

Our study findings have shown that the combination of higher prevalence and lower between cluster variation on sero-positivity compared to parasite positivity may translate into higher study power for serological endpoints and reduced chance of a type 2 error, but should take into account potential differences in effect sizes between metrics. Further research can help to operationally translate serology into a standardised trial tool. There have been only a limited number of studies measuring serology in malaria intervention trials, most of which have been in sub-Saharan Africa (Zambia^43^ and this study in Namibia). While studies in Haiti, Uganda, Zambia, The Gambia and Indonesia have also measured immune responses to Etramp5.Ag1 to assess differences in transmission intensity, further testing of this marker in the context of intervention trials would be particularly useful.

One potential challenge is that rates of antibody acquisition, boost, and decay may differ between antigens and individuals. This may be one reason that strong antigenic signals were not observed for non-Etramp5.Ag1 markers in this study. Sequence variations in parasite proteins between populations or regions may also lead to some variation in antibody responses. A number of studies have observed a reduction in the clonal diversity of the parasite population in areas with decreasing transmission intensity or low rates of importation.^44^^,^^45^ Serological outcomes may be more stable across antigens in these settings, such as our study site in Namibia,^21^ while in higher transmission settings or areas with low transmission but high rates of importation, multi-antigen panels may be beneficial to capture the full breadth of antibody responses across the population. Fortunately, refining the design of recombinant proteins for more precise serological surveillance is the subject of ongoing work.^46^^,^^47^ Improvements in assay design are currently leveraging multiplexing technology to measure the combined response to diverse panels of antigenic variants, capturing the full breadth of antibody responses in a population for single diseases or across multiple pathogens. Other strategies include the production of chimaeric proteins with specific epitopes to these variants.

Biological variations in antibody responses are inevitable to some degree and integrating serological data with other important measures, such as age, can allow a more comprehensive characterisation of epidemiological trends. For standardised surveillance across locations, panels based on studies in a diversity of settings will be beneficial.^48^ Serological surveillance is increasingly being standardised for a number infectious diseases such as dengue,^49^ trachoma,^50^ and lymphatic filariasis.^51^ The inclusion of malaria in multi-disease sero-diagnostic panels^52^^,^^53^ can allow for cost and time efficiencies, reducing the number of single-disease surveys required in diverse health monitoring programmes. This can help guide integrated programme delivery rather than a reliance on multiple vertical programmes delivered separately.

Serological endpoints in intervention trials have become accepted as outcomes for other infectious diseases, such as arboviruses. For example, an ongoing cluster randomised trial on the efficacy of *Wolbachia*-infected *Aedes aegypti* in Brazil will measure reductions in sero-incidence to dengue, zika, and/or chikungunya virus as primary trial endpoints.^54^ In a similar randomised controlled trial in Indonesia, baseline sero-prevalence was used to infer age-specific transmission rates and median age to first infection to inform trial design.^55^ In the case of Zika, it has been suggested that the use of select antigens could help distinguish between antibody responses arising from vaccination versus natural infection, offering advantages over molecular diagnostics for trial evaluation and sampling.^56^ The findings from this study, together with ongoing innovations in assay design and multi-disease platforms, illustrate the potential application of serological markers as endpoints in randomised trials, especially in settings where measures of clinical incidence or infection may be less reliable due to limitations in health care seeking, or incomplete testing and reporting.

## Contributors

LW, MSH, IK, and CD accessed and were responsible for the raw data associated with the study and took the decision to submit the manuscript for publication. MSH, IK, and RG conceptualised and designed the study. MSKD, AB, JLS contributed to study design. MSH and RG provided overall oversight of the study. KWR and HN led the field implementation of the trial. LMP, CSG, and VS supported trial field coordination. LMP, LS, LW, and JY led the cross-sectional survey. PU and SK supported collaboration with the Namibia Ministry of Health and Social Services. KKAT and DM led the laboratory activities. CP, TH, and JB conducted the serological testing. MT and LMP conducted the molecular testing. LMP, BG, and CD provided additional oversight of the laboratory activities. LW led data management and analyses of serological data. BW supported data analyses. IK, MSH, and CD advised on the data analyses. LW wrote the manuscript. All authors contributed to data interpretation and approved the final draft of the manuscript.

## Declaration of interests

MSH declares research grants to her institution from Novartis Foundation, Bill & Melinda Gates Foundation and Horchow Foundation to conduct the cluster randomised trial. IK declares research grants to Wits Health Consortium from the Bill & Melinda Gates Foundation to conduct the cluster randomised trial and to support field visits and attendance at project meetings and scientific conferences related to the trial. DM declares research grants to the University of Namibia from Novartis Foundation and the Bill & Melinda Gates Foundation via a subaward from UCSF. JLS declares receiving salary from the Bill & Melinda Gates Foundation grant that co-funded the study.

## Supplementary materials

Image, application 1

Image, application 2

Image, application 3

Image, application 4
