# Supplementary material for: Serological evaluation of the effectiveness of reactive focal mass drug administration and reactive vector control to reduce malaria transmission in Zambezi Region, Namibia: Results from a secondary analysis of a cluster randomised trial
Source: eClinicalMedicine. 2022 Feb 14;44:101272. doi: 10.1016/j.eclinm.2022.101272 (PMC8851292; doi:10.1016/j.eclinm.2022.101272)
Supplement: Supplementary file 1 [file mmc1.docx]

Figure S1. Number of clusters by study arm. 2x2 factorial design allows for 3 intervention comparisons to be assessed, including RACD (with or without RAVC) vs. rfMDA (with or without RAVC), No RAVC (with or without rfMDA) vs. RAVC (with or without rfMDA), and RACD only vs. rfMDA with RAVC. The table shows the number of clusters for each comparison.

Table S1. Summary of antigen constructs and coupling conditions in multiplex Luminex panel.

Table S2. Demographics of study population by study arm

Figure S2. Sero-prevalence by intervention and antigen. Unadjusted mean sero-prevalence by study arm and intervention, estimated using generalised linear models (log link, binomial family, GEE with clustering at EA-level). Mean sero-prevalence is shown for rfMDA vs RACD (black), RAVC vs. no RAVC (blue) and rfMDA plus RAVC vs. RACD only (magenta)

Table S3. Sero-prevalence by intervention and antigen. Mean sero-prevalence by antigen and intervention, estimated with generalised linear models (log link, binomial family, GEE with clustering at EA-level).

Table S4. Etramp5.Ag1 sero-prevalence ratio by study arm and intervention. Sero-prevalence ratio by study arm and intervention, estimated using generalised linear models (log link, binomial family, GEE with clustering at EA-level).

Figure S3. Distribution of EA-level sero-prevalence for Etramp5.Ag1, by intervention. The distribution of cluster-specific sero-prevalence (shown as kernel density plots) is compared in the rfMDA vs. RACD arms (black), RAVC vs. no RAVC arms (blue) and rfMDA plus RAVC vs. RACD only arms (magenta). Solid and dotted vertical lines indicate the median EA-level sero-prevalence for clusters in the intervention arms (rfMDA, RAVC, or rfMDA + RAVC) and the control arms (RACD, No RAVC, or RACD only), respectively.

Table S5. Prevalence of qPCR-detected infection, (A) as reported in Hsiang et el^8^ and (B) subset of individuals included in serological analysis.

Figure S4. Unadjusted mean AUC value by study arm and intervention, estimated using generalised linear models (log link, gaussian family, GEE with clustering at EA-level). Mean AUC is shown for rfMDA vs RACD (black), RAVC vs. no RAVC (blue) and rfMDA plus RAVC vs. RACD only (magenta)

Table S6. Etramp5.Ag1 AUC ratio by study arm and intervention. Ratio of log AUC values by study are and intervention is estimated using generalised linear models (log link, gaussian family, inverse-weighted by 95%CI AUC) and adjusted for EA incidence in 2016, proportion of EA cases covered, median time to intervention, and distance from villages receiving an MOHSS intervention.

Figure S5. Distribution of EA-level AUCs for Etramp5.Ag1, by intervention. The distribution of cluster-specific AUC values (shown as kernel density plots) is compared in the rfMDA vs. RACD arms (black), RAVC vs. no RAVC arms (blue) and rfMDA plus RAVC vs. RACD only arms (magenta). Solid and dotted vertical lines indicate the median EA-level sero-prevalence for clusters in the intervention arms (rfMDA, RAVC, or rfMDA + RAVC) and the control arms (RACD, No RAVC, or RACD only), respectively.

Figure S6. Etramp5.Ag1 antibody acquisition model fit by enumeration area. Antibody acquisition are fit to estimates of the geometric mean MFI by age group for each antigen and cluster. Using the model fit, total area under the antibody acquisition curve, referred to as the AU value, represents the cumulative antibody response across all ages. AUC values are estimated using an antibody acquisition model fit that is extrapolated to a standardised age range of 1-90 years to account for between cluster variation in age range.

**Table S7. Individual-level association between Etramp5.Ag1 sero-positivity (response variable) and concurrent qPCR-positivity.** Odds ratios are based on generalised linear model with GEE clustering at the EA-level and adjusted for age group (<5 years, 5-15 years, >15 years), gender, fever, study arm, and 2016 EA incidence.

Figure S7. Sero-prevalence compared to qPCR prevalence and clinical incidence rate. Comparison of sero-prevalence vs qPCR prevalence (A) and sero-prevalence vs. clinical incidence (B) are shown on the scatter plot on the log-scale. Each data point represents a study cluster, with the point diameter indicating either qPCR sample size per cluster (>100, 50-100, or <50 individuals) or person years at risk (PYAR) per cluster (>300, 200-300, or <200) and 95%CI for prevalence or incidence represented by the horizontal and vertical lines. Clusters in the RACD only study arm are indicated by the darker points to highlight the range of baseline prevalence and incidence values in the control arms.

**Table S8. Relationship between cluster-level sero-prevalence, qPCR-prevalence, and clinical incidence.** Mean and 95% credible interval (CrI) of posterior from MCMC model fit shown for parameters ⍺_0_ (baseline log odds or log sero-prevalence), 𝛽_0_ (increase in log odds or log sero-prevalence), 𝛽_1,_ 𝛽_2_ (fixed effect of rfMDA, RAVC on rate of change in sero-prevalence per unit increase in qPCR prevalence or clinical incidence) and 𝛽_3_ (interaction effect of rfMDA and RAVC on rate of change in sero-prevalence per unit increase in qPCR prevalence or clinical incidence).
